# Discordant Drug Checking Results and Risk Reduction Behaviours: Findings From the ChecKnow Study in France

**DOI:** 10.1111/dar.70216

**Published:** 2026-07-21

**Authors:** Samuel Tobias, Julio Del Amo, Sophie Albert, Marion Aubert, Anissa Zerrari, Pierre Chappard, Lianping Ti, Perrine Roux

**Affiliations:** ^1^ British Columbia Centre on Substance Use Vancouver Canada; ^2^ School of Population and Public Health University of British Columbia Vancouver Canada; ^3^ Sciences économiques et sociales de la santé & traitement de l’information médicale Marseille France; ^4^ Faculté de Médecine Aix‐Marseille Université Marseille France; ^5^ Association PsychoACTIF Marseille France; ^6^ Department of Medicine University of British Columbia Vancouver British Columbia Canada

**Keywords:** drug checking, France, harm reduction, health belief model

## Abstract

**Introduction:**

Drug checking services (DCS) provide people with information about the contents of their drugs, reducing uncertainty about the composition of substances from unregulated markets. However, their impact on behaviour change has been sparsely evaluated. This study aimed to assess behaviour change following receipt of a drug checking result among users of French DCS.

**Methods:**

Data were drawn from ChecKnow, a French e‐cohort of people who use drugs. Using cross‐sectional, baseline survey data, we identified participants who accessed face‐to‐face DCS within the previous 6 months. Reported drug checking results were categorised as containing only the expected substance, a non‐psychoactive diluent, a psychoactive adulterant, or an entirely different substance. We assessed whether discordance between participants' drug checking result and their expectation was associated with subsequent engagement in risk‐reducing behaviour, using Poisson regression adjusted for relevant confounders.

**Results:**

A total of 1363 participants completed the survey and 480 (35.2%) reported ever using DCS. Compared to results containing only the expected substance, the prevalence of engaging in risk‐reducing behaviour increased with each category: diluted (adjusted prevalence ratio [aPR] = 2.78; 95% confidence interval [CI] 0.84, 9.18), adulterated (aPR = 3.34; 95% CI 1.52, 7.34) and absent (aPR = 5.48; 95% CI 2.61, 11.50).

**Discussion and Conclusions:**

Receipt of unexpected drug checking results was associated with a graded increase in the likelihood of engaging in risk reduction behaviours, with the strongest effects observed when the expected substance was entirely absent. These findings suggest that DCS may effectively promote behavioural adaptation in response to potential drug‐related risks.

## Key Points Summary

- Drug checking has only recently been integrated into French harm reduction programs.
- ChecKnow is an e‐cohort of people who use drugs in France.
- We assessed if drug checking result discordance was associated with behaviour change.
- Unexpected results were associated with a higher likelihood of risk‐reducing behaviours.
- The health belief model helps frame these findings.

## 1 Introduction

Drug checking services provide people who use drugs with timely, individualised information on the composition of substances they intend to consume [1, 2]. By equipping individuals with this information, drug checking aims to promote harm‐reducing behaviours, such as discarding adulterated drugs or lowering doses, thereby reducing drug‐related risks. These efforts are increasingly important in the context of an evolving unregulated (illicit) drug market, characterised by adulteration, new psychoactive substances, and variable potency. Across Europe, this unpredictability, particularly the presence of synthetic adulterants and inconsistent drug contents, has contributed to heightened drug toxicity risks and other drug‐related harms [3].

Prior to the recent escalation in market complexity with the introduction of new psychoactive substances [4, 5], drug checking services had already been established in parts of Europe, with historical roots dating back to the 1960s [1, 6, 7]. While formal drug checking programs have operated in several European countries since the 1990s, their integration into public health infrastructure in France has been limited. Formalised services emerged in France during the early 2000s, but have continued to operate under legal and political scrutiny [8]. To date, there is limited evidence on their behavioural impact in France, where differences in drug market composition, harm reduction infrastructure, and the policy environment may influence both drug checking utilisation and behavioural outcomes. Where evaluations have been conducted internationally, they have largely focused on specific contexts such as nightlife and festival settings [9, 10, 11, 12], supervised drug consumption rooms [13], or regions experiencing acute toxicity crises [14], limiting the generalisability of findings to broader populations. France therefore presents a novel context for evaluation, with newly implemented drug checking services operating at a national level, in the absence of a crisis (e.g., elevated mortality rates due to fentanyl toxicity), and within a distinct harm reduction and policy environment.

While several studies conducted in other settings have reported that unexpected drug checking results are associated with subsequent behaviour change [2, 11, 15], to our knowledge, no studies have examined how varying levels of discordance between expected and detected substances relate to enacted (as opposed to hypothetical) risk reduction behaviours. The health belief model offers a useful theoretical framework for understanding how drug checking results may influence these behaviour changes [15, 16, 17]. The model describes that individuals are more likely to engage in protective health behaviours when they perceive heightened susceptibility to harm, greater severity of potential consequences, and when actionable cues to modify behaviour are present. In the context of drug checking, unexpected or surprising analysis results may increase individuals' perception of risk and serve as cues to action that motivate changes in planned substance use [18]. Despite its relevance, the health belief model has rarely been used to formally guide analyses of how people who use drugs respond to drug checking results, with existing applications largely limited to post hoc interpretation of findings [15].

The objective of this study is to examine the association between drug checking result discordance (i.e., how aligned the drug checking results are with the expectation) and engagement in risk‐reducing behaviour change among people accessing drug checking services in France. We then explore whether increasing levels of discordance between expected and detected substances corresponded to a dose–response relationship in behaviour change, consistent with theoretical constructs of the health belief model. To our knowledge, this is the first national‐level study examining the behavioural impact of drug checking services in France, and one of the first evaluations outside the context of an ongoing drug toxicity crisis.

## 2 Methods

Drug checking services in France can be accessed ‘face‐to‐face’ in some harm reduction associations (Centre d'accueil et d'accompagnement à la réduction des risques pour usagers de drogues) or ‘pop‐up’ style at nightlife events. In these settings, analysis equipment (e.g., thin‐layer chromatography, liquid chromatography with ultraviolet detection) is onsite allowing for immediate analysis and returning of results. For people outside of city centres or by choice, samples can be sent by post to associations that handle analysis by mail, although this is a recent development.

This study was prepared in accordance with the Strengthening the Reporting of Observational Studies in Epidemiology (STROBE) reporting guideline for cross‐sectional studies. Data for this study originated from the baseline, cross‐sectional survey of ChecKnow, an anonymous, longitudinal e‐cohort designed to evaluate the effectiveness of drug checking services in France [19]. An e‐cohort is an online study cohort of participants [20], in this case recruited through social networking and harm reduction websites, as well as promotional materials (i.e., posters) distributed through harm reduction programs and centres across France. The initial baseline surveys were completed between June 2024 and December 2024. Participants self‐recruited and completed the survey online using either a computer or an internet‐enabled mobile device. Eligibility criteria required participants to be 18 years or older and to report illicit substance use. Participants may have previously used drug checking services or not. All participants provided informed consent prior to completing the survey and were offered the opportunity to enter a draw for a €10 gift card. The study was approved by the Ethics Committee of Aix‐Marseille University on 2 May 2023 (n.N/File ref.: 2023‐04‐13‐01 DPO N°569,910). The data protection officer certified the study's compliance with the European Union's General Data Protection Regulation, and with the amended French law of 6 January 1978 on data processing, data files and individual liberties.

The survey included questions on sociodemographic characteristics, substance use patterns, harm reduction engagement, and health and wellbeing indicators. The survey was conducted in French; all survey items were translated into English post hoc for the present analysis. Participants were asked to report their previous use of drug checking services and frequency of engagement: ‘Have you ever had your drugs analysed by an organisation?’ and ‘If yes, how long ago was your last drug check?’ For this study, we restricted the sample to participants who reported accessing face‐to‐face drug checking within the preceding 6 months to minimisze recall bias for the primary exposure and outcome variables. We removed participants who reported only accessing services by mail, as remote drug checking is a nascent service modality in France; we hypothesised the cue to action from an in‐person drug check operates through a different mechanism than in mail‐in drug checking as results are returned after a delay.

### 2.1 Exposure and Outcome Variables

The primary exposure variable was the level of discordance between participants' expectations and the results they received from drug checking. Participants were asked: ‘The last time you had a drug check, did the substance match what you expected?’ Response options included: ‘It was the expected substance’; ‘It was the expected substance but with a different purity than expected’; ‘The expected substance was mixed with a non‐psychoactive cutting agent’; ‘The expected substance was mixed with a psychoactive cutting agent’; ‘It was not the expected substance’; ‘The analysis did not provide a result’; and ‘Other.’ As participants selected only one response, categories were mutually exclusive. Responses were grouped into four ordered categories reflecting increasing deviation from expectation. For analysis, the first two responses were categorised as ‘as expected,’ with the remaining responses grouped into ‘diluted,’ ‘adulterated,’ and ‘not the expected drug,’ reflecting increasing deviation from expectations and theorised gradations in perceived susceptibility and severity consistent with the health belief model; this tiered approach has been previously implemented in drug checking research [21]. No participants selected ‘the analysis did not provide a result’; ‘Other’ responses (*n* = 4) were reviewed and recoded to the most appropriate category based on response content.

The outcome variable was based on participants' self‐reported behaviour change following drug checking, assessed by the question: ‘After that drug check, did you change your consumption behaviour regarding the analysed substance?’ Response options included: ‘No, I consumed the substance as planned’; ‘Yes, I did not consume the substance’; ‘Yes, I saved the substance for another occasion’; ‘Yes, I took a lower dose than planned’; ‘Yes, I took a higher dose than planned’; ‘Yes, I changed my supplier/source’; and ‘Yes, I was worried, and the analysis reassured me.’ For analysis, responses were dichotomised into a binary outcome: engaging in a risk‐reducing behaviour versus not (Table 2). Risk‐reducing behaviours were defined as active modifications to planned substance use intended to reduce exposure to potential harm; saving for a later occasion was not classified as risk‐reducing on the basis that it represents deferred rather than avoided exposure. This classification was developed in consultation with our community advisory board and reflects engagement in harm reduction practices rather than a safety judgement, as maintaining a planned consumption pattern or modifying a dose may, in some contexts, represent appropriate decisions given the information received.

### 2.2 Statistical Analysis

Descriptive statistics were calculated for independent variables, stratified by engagement in risk‐reducing behaviour. Chi‐square tests of independence or Fisher's exact tests were used to compare proportions across strata.

We constructed a multivariable Poisson regression model with a log link and robust (sandwich) standard errors to examine the association between drug checking result concordance and self‐reported risk‐reducing behaviour change. We estimated prevalence ratios rather than odds ratios because the outcome was common. Potential confounders were identified a priori using a directed acyclic graph informed by existing literature on factors associated with both drug checking utilisation and subsequent behaviour change [2, 15, 22, 23]. The directed acyclic graph was developed to represent hypothesised causal relationships between the exposure (drug checking result concordance) and the outcome (behaviour change). A modified disjunctive cause criterion was applied to identify covariates for inclusion, incorporating variables empirically or theoretically associated with the exposure, the outcome or both, provided they were not on the causal pathway [24]. Variables included in the adjusted model were participant age (continuous numeric), gender (man, woman, gender minority [collapsed due to small cell sizes]), education level (less than high school diploma, baccalaureate diploma [high school], undergraduate degree, graduate degree), housing status (own home, staying with family, staying with friends, living in a shelter, alternative housing, non‐stable housing), employment status (work, do not work, on disability, student/apprentice, retired), heroin use patterns over the last month (never, less than weekly, more than weekly), stimulant use patterns over the last month (including cocaine, crack, ecstasy, methylenedioxymethamphetamine [MDMA], speed, cathinones), psychedelic use patterns over the last month (including cannabis, synthetic cannabinoids, hallucinogens, ketamine, and other new psychoactive substances), and drug checking frequency in the past 6 months. Covariate selection was limited to variables available in the ChecKnow survey; therefore, some potentially relevant confounders may not have been captured.

Unadjusted prevalence ratios, adjusted prevalence ratios (aPR) and corresponding 95% confidence intervals (CI) using robust standard errors were reported across levels of drug checking result concordance. Two participants were not included in the multivariable regression due to missing data (one refused a response to education level, and one did not know how many times they had previously used drug checking services). All analyses were conducted using R (version 4.5.0) in RStudio (version 2025.05.1) [25, 26, 27].

To address potential inconsistencies in the temporal sequence between receipt of drug checking results and reported behaviour change, we conducted a sensitivity analysis excluding participants who indicated that their motivation for drug checking was to analyse substances after consumption. Since these cases may reflect scenarios where behaviour change could not plausibly have been influenced by the drug checking result, this restriction allowed us to assess the robustness of the observed associations. The primary multivariable Poisson regression model was run using the restricted sample. In a second sensitivity analysis, we re‐estimated the primary model including participants who accessed drug checking by mail only (*n* = 67), to assess whether their exclusion influenced the observed associations.

## 3 Results

### 3.1 Descriptive Statistics

Our study sample was 32.0% women and 13.3% gender minority; 37.0% reported having an undergraduate degree and 24.3% a graduate one. Regarding substance use characteristics, 84.0% reported some use of stimulants, 81.8% reported some use of psychedelics, and 6.6% reported the use of heroin. Participant demographics are available in Table 1.

**TABLE 1:** Self‐reported participant demographics, stratified by reported risk‐reduction behaviour change following the receipt of most recent drug checking result.

	Overall (*n* = 181)	Risk reduction behaviour change (*n* = 39)	No behaviour change (*n* = 142)
Age (mean and SD)	32.0 (10.1)	32.0 (10.9)	32.0 (9.9)
Gender			
Man	99 (54.7)	20 (51.3)	79 (55.6)
Woman	58 (32.0)	15 (38.5)	43 (30.3)
Trans man	4 (2.2)	2 (5.1)	2 (1.4)
Trans woman	5 (2.8)	0 (0.0)	5 (3.5)
Non‐binary	11 (6.1)	2 (5.1)	9 (6.3)
Other	3 (1.7)	0 (0.0)	3 (2.1)
I don't know	1 (0.6)	0 (0.0)	1 (0.7)
Sexual orientation			
Heterosexual	84 (46.4)	17 (43.6)	67 (47.2)
Homosexual	27 (14.9)	11 (28.2)	16 (11.3)
Bisexual	38 (21.0)	3 (7.7)	35 (24.6)
Pansexual	20 (11.0)	6 (15.4)	14 (9.9)
Asexual	2 (1.1)	0 (0.0)	2 (1.4)
Other	3 (1.7)	0 (0.0)	3 (2.1)
I don't know	5 (2.8)	2 (5.1)	3 (2.1)
Refused	2 (1.1)	0 (0.0)	2 (1.4)
Education			
High school	37 (20.4)	9 (23.1)	28 (19.7)
Some high school	32 (17.7)	9 (23.1)	23 (16.2)
Undergraduate	67 (37.0)	16 (41.0)	51 (35.9)
Graduate	44 (24.3)	5 (12.8)	39 (27.5)
Refused	1 (0.6)	0 (0.0)	1 (0.7)
Housing			
Own home	132 (72.9)	28 (71.8)	104 (73.2)
Staying with family	11 (6.1)	2 (5.1)	9 (6.3)
Staying with friends	11 (6.1)	1 (2.6)	10 (7.0)
Organisation	5 (2.8)	1 (2.6)	4 (2.8)
Alternative housing	9 (5.0)	3 (7.7)	6 (4.2)
Non‐stable	13 (7.2)	4 (10.3)	9 (6.3)
Municipality population			
< 500	6 (3.3)	2 (5.1)	4 (2.8)
500–3000	18 (9.9)	3 (7.7)	15 (10.6)
3000–20,000	19 (10.5)	5 (12.8)	14 (9.9)
20,000–50,000	16 (8.8)	5 (12.8)	11 (7.7)
50,000–100,000	22 (12.2)	4 (10.3)	18 (12.7)
> 100,000	83 (45.9)	15 (38.5)	68 (47.9)
I don't know	16 (8.8)	5 (12.8)	11 (7.7)
Refused	1 (0.6)	0 (0.0)	1 (0.7)
Employment			
Working	102 (56.4)	18 (46.2)	84 (59.2)
Not working	43 (23.8)	10 (25.6)	33 (23.3)
Disability	12 (6.6)	4 (10.3)	8 (5.6)
Student/apprentice	23 (12.7)	7 (17.9)	16 (11.3)
Retired	1 (0.6)	0 (0.0)	1 (0.7)
Heroin use			
Never	169 (93.4)	35 (89.7)	134 (94.4)
< Weekly	4 (2.2)	1 (2.6)	3 (2.1)
> Weekly	8 (4.4)	3 (7.7)	5 (3.5)
Stimulant use			
Never	29 (16.0)	5 (12.8)	24 (16.9)
< Weekly	71 (39.2)	13 (33.3)	58 (40.8)
> Weekly	81 (44.8)	21 (53.8)	60 (42.3)
Psychedelic use			
Never	33 (18.2)	8 (20.5)	25 (17.6)
< Weekly	50 (27.6)	6 (15.4)	44 (31.0)
> Weekly	98 (54.1)	25 (64.1)	73 (51.4)
Drug checking frequency (past 6 months)			
1 time	51 (28.2)	10 (25.6)	41 (28.9)
2–4 times	89 (49.2)	22 (56.4)	67 (47.2)
5–10 times	26 (14.4)	5 (12.8)	21 (14.8)
> 10 times	14 (7.7)	2 (5.1)	12 (8.5)
I don't know	1 (0.6)	0 (0.0)	1 (0.7)
Drug checking result			
Expected	126 (69.6)	14 (35.9)	112 (78.9)
Diluted	15 (8.3)	5 (12.8)	10 (7.0)
Adulterated	19 (10.5)	9 (23.1)	10 (7.0)
Not expected	21 (11.6)	11 (28.2)	10 (7.0)

*Note:* Data sourced from the baseline ChecKnow survey in France.

**FIGURE 1:**
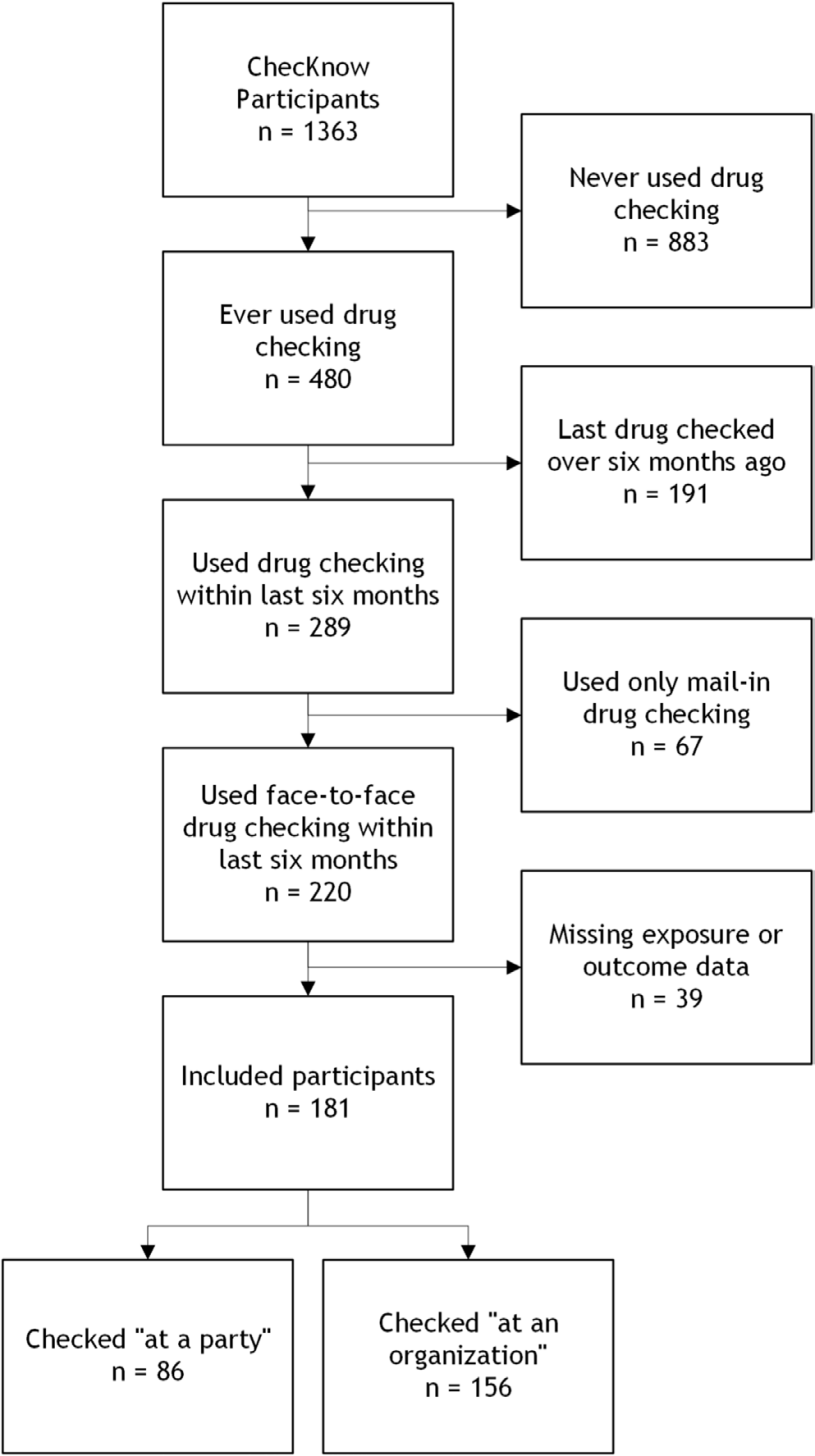
Flowchart depicting participant eligibility criteria. Data sourced from the baseline survey of the ChecKnow e‐cohort in France.

Of the 1363 individuals who completed the baseline ChecKnow survey, 883 (64.8%) had never used drug checking services and were excluded. Of the remaining 480 (35.2%) who had ever used drug checking, 191 (39.8%) had last done so more than 6 months prior and were excluded to minimise recall bias. Of the 289 who had used drug checking within the past 6 months, 67 (23.2%) had accessed services by mail only and were excluded, as remote drug checking is a nascent service modality in France. A further 39 participants were excluded due to missing exposure or outcome data, leaving a final analytic sample of 181 participants (13.3% of the total baseline survey sample; Figure 1).

Of the 181 participants in our sample, 39 (21.5%) reported engaging in a risk reduction behaviour following the receipt of their drug checking results (Table 2). The total sample was comprised of 126 (69.6%) participants whose drugs met their expectation, 15 (8.3%) whose were diluted, 19 (10.5%) whose were adulterated with a psychoactive agent, and 21 (11.6%) whose were not the expected substance.

**TABLE 2:** Self‐reported risk reduction behaviour changes of ChecKnow participants following receipt of drug checking results and recoding for binary outcome classification (*n* = 181).

Survey question: After the drug check, did you change your consumption behaviour regarding the analysed substance?		
Survey response option	Outcome classification	*n* (%)
No, I consumed the substance as planned	No risk reduction behaviour change	118 (65.2)
Yes, I did not consume the substance	Risk reduction behaviour change	16 (8.8)
Yes, I saved the substance for another occasion	No risk reduction behaviour change	8 (4.4)
Yes, I took a lower dose than planned	Risk reduction behaviour change	16 (8.8)
Yes, I took a higher dose than planned	No risk reduction behaviour change	4 (2.2)
Yes, I changed my supplier/source	Risk reduction behaviour change	7 (3.9)
Yes, I was worried, and the analysis reassured me	No risk reduction behaviour change	12 (6.6)

### 3.2 Association Between Result Discordance and Risk Reduction Behaviour

In the unadjusted model, increasing levels of drug checking result discordance were associated with higher prevalence of reporting risk‐reducing behaviour change. Compared to participants whose drug checking result matched their expectation, the prevalence of risk‐reducing behaviour changes increased among those who received a result indicating dilution, adulteration, or complete absence of the expected drug (Table 3). In the adjusted model accounting for sociodemographic and substance use factors, the estimates for adulterated and absence of expected drug remained significant, and all three point estimates followed a dose–response pattern. Compared to participants who received the expected drug, those who received a diluted result had 2.78‐times the prevalence of risk‐reducing behaviour change (95% CI 0.84, 9.18). The prevalences were higher among those who received a psychoactive adulterant result (aPR = 3.34; 95% CI 1.52, 7.34) and highest among those whose expected drug was absent (aPR = 5.48; 95% CI 2.61, 11.50).

**TABLE 3:** Associations between drug checking result discordance and risk‐reducing behaviour change among ChecKnow participants in France (*n* = 179).

Drug checking result	PR (95% CI)	*p*	aPR (95% CI)^a^	*p*
As expected	Reference		Reference	
Non‐psychoactive diluent	3.00 (1.26, 7.16)	0.013	2.78 (0.84, 9.18)	0.093
Psychoactive adulterant	4.26 (2.15, 8.45)	< 0.001	3.34 (1.52, 7.34)	0.002
Expected drug not present	4.71 (2.49, 8.95)	< 0.001	5.48 (2.61, 11.50)	< 0.001

Abbreviations: aPR, adjusted prevalence ratio; CI, confidence interval; PR, prevalence ratio.

^a^ Model adjusted for age, gender, education level, housing status, employment status, last month heroin use, last month stimulant use, last month psychedelic use, and last six‐month drug checking frequency.

### 3.3 Sensitivity Analyses

A total of 58 participants (32.0%) reported that their primary motivation for drug checking was to analyse their substance after consuming it. In a sensitivity analysis excluding these individuals (n remaining = 121), the dose–response relationship between increasing discordance in drug checking results and engagement in risk‐reducing behaviour change remained consistent with the primary model. Compared to participants whose drug checking result matched their expectations, the prevalence of behaviour change was higher among those receiving a diluted result (aPR = 3.46; 95% CI 0.74, 16.12), a psychoactive adulterant result (aPR = 4.76; 95% CI 1.43, 15.85), and were highest among those whose expected drug was absent (aPR = 6.38; 95% CI 1.59, 25.63).

In a sensitivity analysis including mail‐in drug checking participants originally excluded, the dose–response relationship was unchanged, with adjusted prevalence ratios closely matching the primary model (diluted aPR = 2.79; 95% CI 0.86, 9.11; psychoactive adulterant aPR = 3.26; 95% CI 1.52, 7.01; expected drug absent aPR = 4.97; 95% CI 2.40, 10.27).

## 4 Discussion

In this study of people who accessed drug checking services in France, we found that discordance between expected and detected substances was associated with self‐reported risk‐reducing behaviour change. Participants who received results indicating dilution with a non‐psychoactive filler, psychoactive adulteration, or complete absence of the expected drug demonstrated progressively higher prevalence of modifying their consumption behaviour compared to those whose drug check confirmed the expected substance. Although the confidence intervals overlapped, the point estimates followed a clear dose–response pattern, with the greatest likelihood of behaviour change observed among participants whose expected drug was entirely absent. The associations for the two highest levels of discordance remained significant after adjusting for sociodemographic and substance use characteristics.

The observed dose–response pattern aligns with key constructs of the health belief model, which theorises that individuals engage in protective behaviours when they perceive heightened susceptibility to harm and greater severity of potential outcomes [16, 17]. In our study, increasing discordance between the expected and detected substances appeared to serve as a progressively stronger cue to action. Participants who received results indicating psychoactive adulteration or complete absence of the expected substance demonstrated the highest likelihood of behaviour change, consistent with greater perceived risk associated with these findings. In contrast, non‐psychoactive diluents, while still associated with risk reduction behaviour changes, may have been perceived as lower‐risk findings relative to psychoactive adulterants or entirely unexpected substances. These findings suggest that drug checking services may function not only as informational tools but also as behavioural interventions that modify risk perceptions and activate protective behaviours through multiple components of the health belief model, including perceived susceptibility, severity, and cues to action [16].

These results are consistent with prior studies conducted in other settings, which have similarly demonstrated that individuals receiving unexpected or concerning drug checking results are more likely to modify their consumption behaviours [2, 9, 10, 11, 12, 14, 15]. Our study builds on this literature in several important ways. First, it is the first national‐level evaluation of drug checking services in France, capturing a large and diverse population of people who use drugs across multiple regions. Second, the study was conducted in the context of an unregulated drug market that, while affected by new psychoactive substances and adulteration, is generally considered less adulterated than markets in North America or certain other European countries [6, 28]. National forensic surveillance indicates that cocaine purity has risen steadily (averaging 74.3% in 2024) while cutting‐agent adulteration has declined, whereas heroin, though frequently adulterated, is cut predominantly with predictable diluents such as caffeine and paracetamol rather than unexpected psychoactive substances [29]. Consistent with a relatively lower‐discordance market, a French drug checking evaluation reported that only 5.1% of collected samples did not correspond to the declared product [4]. Observing a graded association between increasing levels of discordance and enacted risk‐reducing behaviour change in this relatively lower‐adulteration context strengthens the evidence that drug checking can produce meaningful behavioural change beyond settings with acute drug toxicity crises. Finally, by applying the theoretical framework of the health belief model, we provide conceptual support for these observations, suggesting that even modest discordance between expected and detected substances can act as a cue to action with significant public health benefits. These findings add important evidence from France, where formal drug checking services have only recently been integrated into harm reduction programming and continue to evolve under active legal and policy consideration.

This study has several strengths, including the use of primary data from individuals directly accessing drug checking services, measurement of enacted behaviour change outcomes, and the application of a theory‐informed confounder selection process guided by a directed acyclic graph. Nonetheless, several limitations warrant consideration. First, because the survey was cross‐sectional and based on self‐report, temporality between receipt of drug checking results and subsequent behaviour change cannot be definitively established for all participants. However, the survey questions were phrased to reflect temporality, and sensitivity analyses excluding individuals who reported motivations to check substances after consumption yielded consistent results, supporting the robustness of the observed associations. Second, the use of a self‐recruited sample may limit generalisability to broader populations of people who use drugs, particularly outside of France. Third, regarding the exposure ascertainment, the survey instrument did not capture which drug was checked by the participant, nor what the adulterant being reported on was, nor the mode of analysis (i.e., infrared spectroscopy, colourimetric reagents, etc.). Although the specific substance checked was unknown, participants reported predominantly stimulant (84.0%) and psychedelic (81.8%) use, with relatively low heroin use (6.6%). The behavioural responses observed therefore most likely reflect drug checking of stimulants and psychedelics and may generalise less readily to opioid or benzodiazepine checking, where the risk profile, motivations for checking, and behavioural options may differ. Additionally, while the model adjusted for key sociodemographic and substance use characteristics, residual confounding remains possible due to unmeasured factors not captured in the survey instrument. Finally, because both the exposure and outcome were self‐reported at a single time point, the observed associations are susceptible to recall bias. Beyond the general recall accuracy, which we sought to limit by restricting the sample to participants who accessed drug checking within the preceding 6 months, the retrospective reporting of exposure and outcome introduces the possibility of differential recall, whereby participants' memory of one may be influenced by the other. Correlated recall could tend to bias associations away from the null, potentially overstating the observed dose–response relationship. While our findings are consistent with the theoretically grounded mechanism of the health belief model and with prior studies [10, 12, 15], a future direction would be prospective data linking results to subsequently observed behaviour to confirm the magnitude and direction of these associations. Self‐reported behaviour change may additionally be subject to social desirability bias. Our findings suggest that drug checking services may promote risk‐reducing behaviour change by providing individuals with personalised information that alters perceived risk, consistent with the health belief model, in a setting without an acute drug toxicity crisis. While our findings demonstrate how drug checking results may influence risk‐reducing behaviours among service users, future research should apply the health belief model to better understand the motivations and perceived barriers among people who do not access drug checking services. Exploring these dynamics could inform strategies to expand the reach and accessibility of drug checking to populations who may benefit the most from accessing them. As drug checking services continue to expand and evolve alongside the changing illicit drug supply, our findings show how they contribute to a comprehensive suite of harm reduction and public health interventions aimed at mitigating substance use‐related risks and improving health outcomes for people who use drugs.

## Author Contributions

S.T., L.T., and P.R. conceived of this study. S.T. performed the formal analysis, assisted by J.D.A. S.T. prepared the original version of the manuscript, and all other authors provided review and critical feedback. Each author certifies that their contribution to this work meets the standards of the International Committee of Medical Journal Editors.

## Funding

This work was supported by Institut pour la Recherche en Santé Publique.

## Conflicts of Interest

The authors declare no conflicts of interest.

## Data Availability

The data that support the findings of this study are available on request from the corresponding author. The data are not publicly available due to privacy or ethical restrictions.
